# British Health Stations.—II

**Published:** 1904-02-20

**Authors:** 


### BRITISH HEALTH STATIONS.—II.

(BY OUR SPECIAL MEDICAL COMMISSIONER.)

(*Continued from page* 361.)

### WORTHING.

WORTHING, sometimes described by its ardent admirers as the "Madeira of England," has been noted for its health-giving properties since the middle of the eighteenth century. Fashion and professional favour led to its rapid growth in the early portion of last century. It is now fully established as an excellent resort where sea-air, bathing, and good exposure to sunlight may be enjoyed. Much has been said respecting its peculiar advantages for the invalid.[1] It is well protected on the north by the range of the South Downs, sheltered somewhat by Beachy Head on the east; and although open to the prevailing south and south-west winds its south aspect secures its right to the designation "sunny Worthing."

Meteorological returns [2] go far to show that its climatic conditions are particularly equable, and hence highly favourable for the invalid. It is in great measure "a garden town," and much fruit is grown in the immediate vicinity. The sylvan features are characteristics of much service to health-seeking visitors. The water is excellent, being obtained direct from the hills. The drainage is good. The town is well kept; it is clean, well watered, and in great part lit by electric light. Many parks and pleasure-grounds are available. The extensive sweep of the parade, with its excellent shelters, forms perhaps the chief of its hygienic advantages. It is to be regretted that at low tide the smell from the sea-weed is sometimes objectionable. There is a well-equipped pier, 1,000 feet long and 30 feet wide, which forms an admirable resort for many convalescents and invalids.

The mildness of the winter, and the many attractions offered by the place, have led to the rapid growth of a residential district where many elderly and enfeebled people make their home. The immediate neighbourhood is rich in places of historic and artistic interest. Invalids will find no lack of opportunities for drives through picturesque country. Worthing deserves to take a high place among our winter havens. It has won a good reputation as a beneficial resort for many lung affections. For those phthisical patients who find the sea-air soothing, the equable climate and sunny aspect offer particularly good opportunities for the enjoyment of open-air treatment. Cases of chronic bronchitis, and some patients with asthma, gain much relief by a residence at Worthing. Subjects of chronic rheumatism and renal disease are said to do well. But probably Worthing offers its chief attractions to aged persons, children, invalids, especially those returned from hot climates, convalescents, and weary workers desirous of peace and quiet recreation.

The town is well supplied with hotels, boarding-houses, modern shops, and many excellent medical practitioners. The population is returned at 20,006.

Worthing is an admirable "sanatorium" for the metropolis. It is 61 miles from London, and can be reached by the L. B. and S. C. Railway in 1½ hour.

Mr. George W. May has recently issued for the Corporation of Worthing an excellent little handbook,[3] which medical men intending to send patients to this South of England seaside winter station might consult with advantage.

[1] "On the Climate of Worthing: Its Remedial Influence in Diseases, especially of the Lungs." By W. Goodyer Barker, M.B. (Second edition. London: 1867.)

[2] "The Climates and Baths of Great Britain." Vol. I. P. 380. (London: 1895.)

[3] "Worthing." The Health Resorts Association, 21 St. Bride Street, E.C.

